# CavBench: A benchmark for protein cavity detection methods

**DOI:** 10.1371/journal.pone.0223596

**Published:** 2019-10-14

**Authors:** Sérgio Dias, Tiago Simões, Francisco Fernandes, Ana Mafalda Martins, Alfredo Ferreira, Joaquim Jorge, Abel J. P. Gomes

**Affiliations:** 1 Instituto de Telecomunicações, Delegação da Covilhã, Covilhã, Portugal; 2 Universidade da Beira Interior, Departamento de Informática, Covilhã, Portugal; 3 INESC-ID, Lisboa, Portugal; 4 Universidade de Lisboa, IST, Lisboa, Portugal; 5 Universidade Europeia, Lisboa, Portugal; Universita degli Studi di Roma Tor Vergata, ITALY

## Abstract

Extensive research has been applied to discover new techniques and methods to model protein-ligand interactions. In particular, considerable efforts focused on identifying candidate binding sites, which quite often are active sites that correspond to protein pockets or cavities. Thus, these cavities play an important role in molecular docking. However, there is no established benchmark to assess the accuracy of new cavity detection methods. In practice, each new technique is evaluated using a small set of proteins with known binding sites as ground-truth. However, studies supported by large datasets of known cavities and/or binding sites and statistical classification (i.e., false positives, false negatives, true positives, and true negatives) would yield much stronger and reliable assessments. To this end, we propose *CavBench*, a generic and extensible benchmark to compare different cavity detection methods relative to diverse ground truth datasets (e.g., PDBsum) using statistical classification methods.

## Introduction

Modeling protein-ligand interactions is crucial to drug discovery and design, as well as to understand bio-molecular structures. While extensive efforts have been applied for many years into discovering new methods to model protein-ligand interactions, a comprehensive mechanism to compare and assess such methods (and algorithms) is still lacking. This makes it difficult to properly ascertain the contributions of each method in the context of the myriad of approaches developed over the past decades.

In the present work, we are particularly interested in benchmarking protein cavity (or pocket) detection methods against one or more databases of cavities (e.g., PDBsum [1, 2]) or even databases of already-known binding sites (e.g., scPDB [3]). Often, these already-known protein binding sites correspond to protein cavities, so they may also work as ground-truth cavities. This explains why detecting pockets/cavities on protein surfaces is an important first step toward identifying protein binding sites for small molecules or ligands [4–6]. Thus, pocket detection plays an important role in protein-ligand docking and structure-based drug design.

Many protein cavity detection methods have been proposed in the literature for the last 35 years (see, for example, [4, 7–17]). However, only a few times have we seen such methods validated or certified relatively to a ground-truth underlying a database of known cavities or a database of known binding sites. This is largely because the first such databases only made their appearance in the early 2000s, after the debut of DIP (Database of Interacting Proteins) available at http://dip.doe-mbi.ucla.edu/ for protein-protein interactions [18], in particular BIND (Biomolecular Interaction Network Database) at https://bio.tools/bind [19] and BindingDB (https://www.bindingdb.org/bind/index.jsp) [20, 21] for interactions between any two molecules consisting of proteins, nucleic acids, and ligands.

Since then, several databases exclusively dedicated to known protein-ligand bindings have been reported in the literature, namely PDBsite [22], PLD [23], SitesBase [24], Binding MOAD [25, 26], FireDB [27], PoSSuM [28], ccPDB [29], Pocketome [30], BioLiP [31], sc-PDB-Frag [32], but only a few have been worked out as ground-truth datasets to certify the accuracy of protein cavity detection methods [33–39], namely SCOP [40], Relibase [41], PDBbind [42, 43], sc-PDB [3], LigASite [44], MPStruc (http://blanco.biomol.uci.edu/mpstruc/). These ground-truth based methods use statistical analysis to check a technique’s accuracy in finding putative binding sites. To this end, they use performance metrics such as precision and recall [45], which are expressed in terms of false positives, true positives, false negatives, or true negatives.

To the best of our knowledge, despite the aforementioned databases of known protein-ligand binding sites and methods to identify protein cavities (or putative binding sites), there is no benchmarking software to compare ground-truth datasets of binding sites. Neither are there approaches to assess cavity detection methods against those ground-truth repositories using statistical classification metrics, including recall, precision, and F-score. We designed CavBench to fulfill this need, and more importantly, we made it XML-extensible to accommodate new cavity datasets, new binding-site datasets, and new cavity detection methods as they become available. At the present time, CavBench includes a single ground-truth dataset of cavities concerning 660 apo proteins and 1633 holo proteins, in a total of 2293 proteins. Holo-proteins consider the structure of each protein combined with their ligand(s), while apo-proteins only consider their isolated form. This dataset is here called *CavDataset*, and combines the clefts, pores, and tunnels retrieved from PDBsum [1, 2]; PDBsum clefts were obtained using SURFNET [46], while PDBsum pores and tunnels were retrieved though Mole [47]. CavBench also integrates the following cavity detection methods: Fpocket [48], GaussianFinder [6], GHECOM [36], and KVFinder [15]. This integration is made possible due to CavBench’s XML-compliance.

In short, the main features of CavBench include platform-independence, interoperability, and extensibility. It is platform-independent since it runs on any major operating system namely, Mac OSX, Unix/Linux, and Windows. Its interoperability stems from its programming in shell scripting and XML interfaces to datasets and methods. Finally, its extensibility also results from its XML-based back-end.

## Materials and methods

### Background

**Fig 1:**
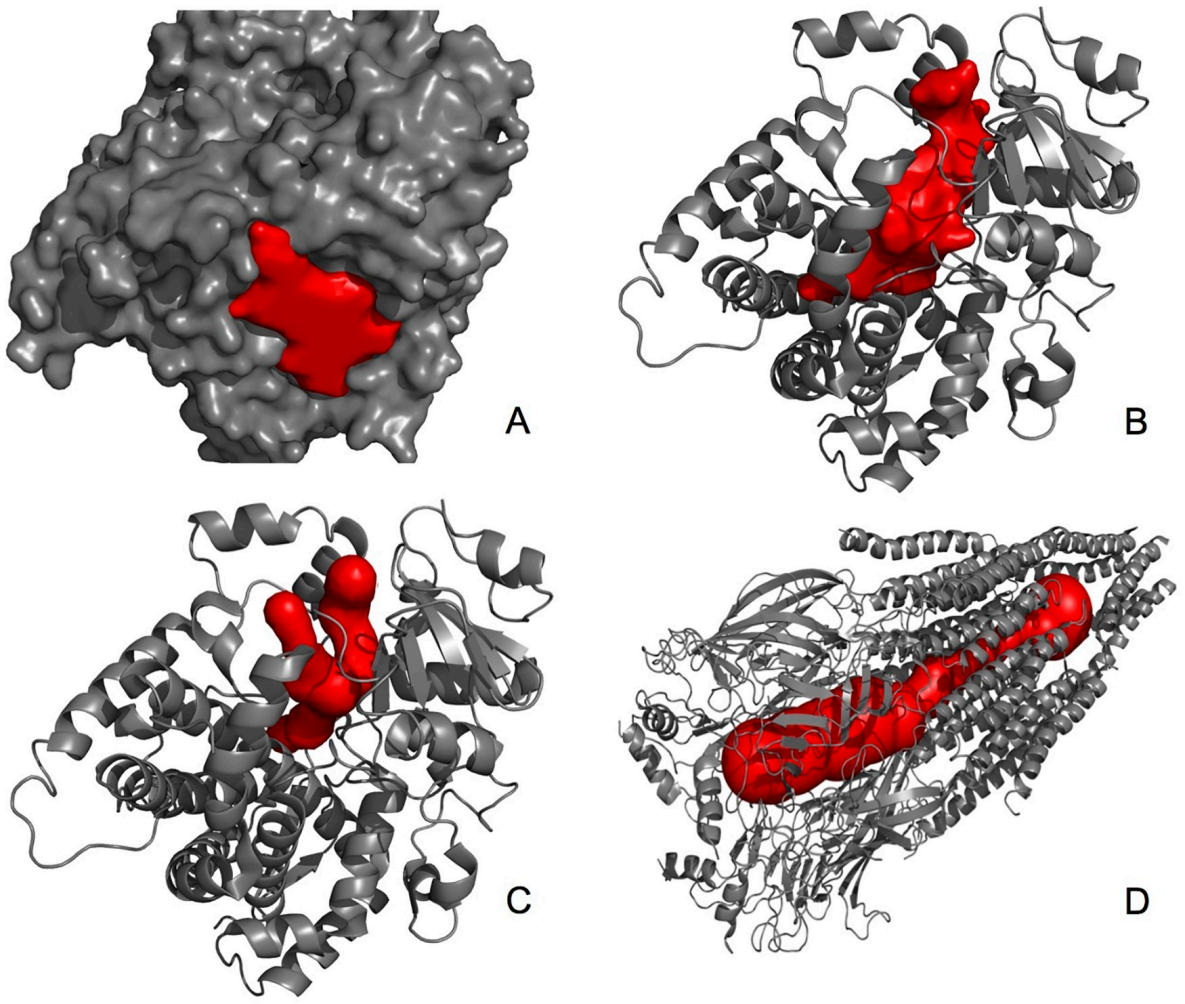
Main types of cavities: A—Pockets, B—Cavities, C—Tunnels, D—Pores (courtesy of Sehnal et al. [49]).

Following the terminology of PDBSum [1, 2], the protein cavities can be classified as *clefts*, *tunnels* (channels), and *pores* (see Fig 1). A cleft is a depression on the molecular surface, and is called a pocket if it is a shallow depression. A cleft often works as binding site for ligands and other proteins. A protein may also possess internal cavities, also called voids, which are isolated from the exterior environment. Voids often are enzymatic reaction sites, as a void constitutes a highly controlled environment inside the protein. But, if a void communicates with the exterior environment of a given protein via one or more tunnels, it is called a chamber. A tunnel is a ligand accessible pathway that goes from the protein surface to a chamber. But, while a tunnel has a single entry on the protein surface, a pore has two entries (or an entry and an exit). In fact, a pore is a tunnel through the protein that connects an entry to an exit on the protein surface. Many pores work as selective transport pathways across membranes.

### CavBench architecture overview

CavBench has been designed as a layered benchmark. It consists of the following three workspaces (or layers):

- *Ground-truth datasets*. Currently, the first workspace consists of a single dataset of cavities, called CavDataset, but more datasets may be added in the future (e.g., the dataset of cavities concerning already-known binding sites of sc-PDB [3]). In order to incorporate another ground-truth dataset in the first workspace, we need to produce a XML-specification file that describes it; for example, the CavDataset is described by the file CavDataset.xml. Such XML file is generated from a dataset-specific parser (using some programming or scripting language).
- *Method-specific parsers*. The second workspace comprises the parsers that are specific to cavity detection methods. A method-specific parser transforms the output (predicted cavities in each protein) of each method into clusters of spheres described in a XML file.
- *Cavity benchmark*. The third workspace has been designed to contrast the method-specific predicted cavities of one or more proteins against the ground-truth cavities. The benchmark generates not only a cavity overlapping matrix for each protein, which determines the amount of overlapping between ground-truth cavities and method-specific predicted cavities, but also method-specific statistics for true positives, false positives, true negatives, and false negatives, as well as precision, recall, and F-score values for distinct methods.

We proceed by providing a more detailed description of each workspace in CavBench.

### Ground-truth datasets

In order to carry out testing in a reasonable time, the ground-truth workspace comprises a single dataset, called *CavDataset*, which consists of a subset of the PDBsum dataset (http://www.ebi.ac.uk/pdbsum/) [1, 2]. At our best knowledge, PDBsum is the only repository that includes a dataset of geometric cavities of proteins; these cavities do not necessarily match binding sites on protein surfaces. As mentioned above, CavDataset comprises 2293 proteins, among which we find 660 *apo* proteins and 1633 *holo* proteins. An *apo* structure is considered to be an isolated protein without any ligands attached. On the other hand, an *holo* structure is a protein-ligand complex, identified by a different PDB entry, and very frequently characterized by some level of structural deformation (in relation to its *apo* form) caused by the binding event.

**Fig 2:**
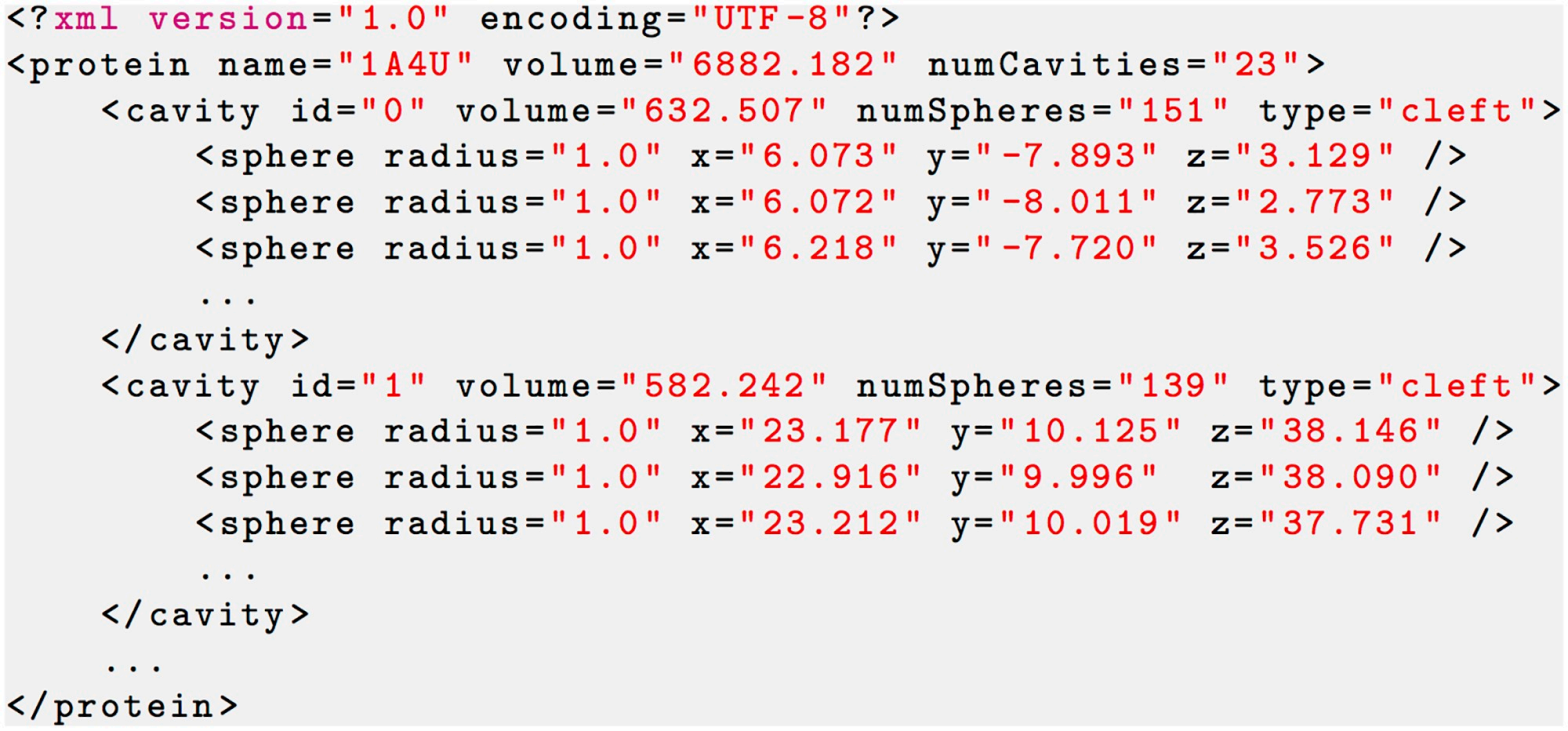
A snippet of XML file 1a4u.xml partially describing two clefts of the protein 1A4U in the CavDataset.

Taking into consideration the classification of protein cavities above (see Fig 1), each protein of the ground-truth dataset is associated to a single .xml file that describes such cavities. For example, the cavities of the protein 1A4U are specified in the file 1a4u.xml of the CavDataset (see Fig 2). This .xml file is the result of the fusion of three separate files: 1a4u-clefts.pl, 1a4u-tunnels.xml, and 1a4u-pores.xml. The first is a Perl file that was directly retrieved from the PDBsum web site, and only describes the clefts of the protein 1A4U. The second and third files describe tunnels and pores as generated from Mole (http://mole.chemi.muni.cz/web/index.php) [47], which is a program that locates and characterizes tunnels and pores in proteins. Note that it is also feasible to directly retrieve a list of tunnels and pores of each protein from PDBsum, which also uses Mole for such purpose, but not their constituent pseudo-atoms and volumes. Mole builds upon a Voronoi tessellation and a Dijsktra path search algorithm to find such types of cavities.

#### PDBsum clefts

**Fig 3:**
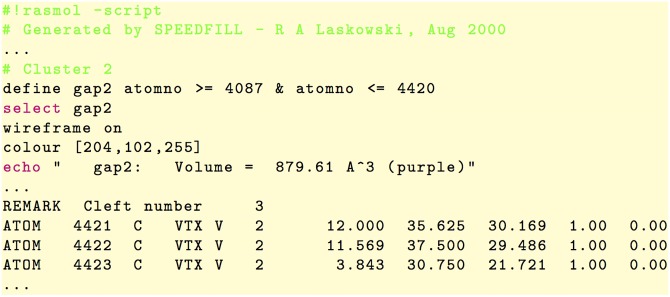
Snippets of the Perl file 1gfs.pl (concerning the protein 1GFS) that describes the volume of the second cluster (or cleft) and three of its dummy atoms.

To generate the .xml files that describe the clefts of the proteins of the CavDataset, first their .pl files (Perl files) were extracted from the PDBsum web site. Each .pl file (without protein structure) lists the dummy atoms (also called pseudo-atoms or hetero-atoms) of each cleft, as illustrated in Fig 3. Then, each .pl file was parsed to generate an .xml file that describes the clefts of each protein. As a result, we obtain the corresponding .xml files for clefts, each one having the structure shown in Fig 2. Note that each cleft is represented by a list of dummy atoms (or spheres), each one specified by means of its radius and center coordinates. As in the PDBsum web site, the clefts in Fig 2 are listed in the decreasing order of their volumes.

#### Mole tunnels and pores

PDBsum provides the tunnels and pores of a given protein, but does not list their dummy atoms and volumes. However, we can get such dummy atoms using Mole (http://mole.chemi.muni.cz/web/index.php). Mole takes the standard PDB file of such a protein and generates two separate .xml files, the first describing its tunnels, while the second its pores.

In practice, because we are only interested in shape information of cavities, our parsers for tunnels and pores only extract geometric cavity information (i.e., their dummy atoms) from a Mole .xml file, generating two CavBench .xml files with the same structure as the one for clefts shown in Fig 2, the first for tunnels and the second for pores. Recall that, as mentioned above, the three .xml files for clefts, tunnels, and pores concerning a single protein are merged into a single .xml file. That is, the CavDataset consists of 2293 .xml files concerning 2293 proteins.

### Method-specific parsers

**Fig 4:**
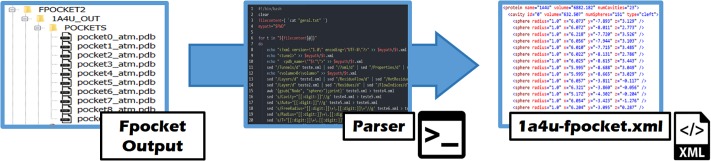
Workflow of the Fpocket-specific parser for the protein 1A4U, that transforms the multiple-file PDB-like output of Fpocket into a single .xml file called 1a4u-fpocket.xml.

These parsers form the second workspace (or layer). As known, the input format of each cavity detection method/software is standard, as it consists of a PDB file describing the structure of a protein. However, its output (e.g., clusters of points) is not standard and varies from a method to another. To standardize the output produced by each cavity detection method, the corresponding method-specific output file (e.g., PDB-like file) must be converted into an XML file describing the cavities of each protein, as shown in Fig 2; that is, each cavity must be expressed as a cluster of spheres, each representing a pseudo-atom. Such PDB-like-to-XML parsing process is illustrated in Fig 4 for a cavity detection method called Fpocket.

Thus, adding a new cavity detection method to CavBench requires:

- To create its PDB-like-to-XML parser.
- To add its dataset to CavBench; that is, to add the .xml files produced by its parser for the proteins existing in CavBench.

In other words, CavBench does not run the code of any specific cavity detection method. However, it incorporates the 2293 .xml files generated by each method-specific parser, one file per protein. Although each .xml file is generated from the typical output PDB-like format file of most methods, each method has its own peculiarities, namely:

#### Fpocket

This is a Voronoi tessellation method [48]. Its output is not a single file, but a set of files for each protein. Fpocket outputs two PDB-like files per cavity. The first file is a .pqr file that lists Voronoi balls (of variable radius) which fill in the cavity. The second file owns extension .pdb and lists the atoms that enter in contact with the Voronoi balls. Therefore, as illustrated in Fig 4, our Fpocket-specific parser reunites all these PDB-like files into a single CavBench’s XML file that describes all cavities of a single protein.

#### GHECOM

GHECOM is a grid-and-sphere method [36] which outputs a single PDB-like file that lists the pseudo-atoms of all cavities of a specific protein. Each pseudo-atom (or hetero-atom) is a sphere whose center is a grid point located outside the protein, with its radius taking on the value of half the spacing. Unlike Fpocket, this method does not provide any information concerning atoms of the protein surface that interface with cavity hetero-atoms. So, our GHECOM-specific parser basically converts the GHECOM output PDB-like file into a CavBench’s XML file that lists the cavities of a given protein and their hetero-atoms.

#### KVFinder

KVFinder is another grid-and-sphere method that outputs a single PDB-like file per protein [15]. As usual, each pseudo-atom is described by its center and radius. However, the listing of pseudo-atoms does not include information about their cavities. To overcome this problem, we used the DBSCAN clustering algorithm [50] to transform the soup of pseudo-atoms outputted by KVFinder into a set of clusters of pseudo-atoms, with each cluster featuring a cavity. Unlike k-means clustering [51, 52], DBSCAN has the advantage of not requiring the *a priori* specification of the number of clusters in the point data. Isolated points or points with only a few neighboring points were considered as noise outliers. Finished such clustering, our KVFinder-specific parser produced the CavBench’s XML file that describes the cavities of each protein in terms of its pseudo-atoms.

#### GaussianFinder

GaussianFinder is a grid-and-surface method [6]. It also produces a single PDB-like file per protein. Similar to KVFinder, it outputs the set of grid nodes concerning cavities, but does not provide the cavity identifier associated to each grid node. Similar to GHECOM, the radius of the pseudo-atom centered at each grid node is half the grid spacing. The cluster of hetero-atoms concerning each cavity was also obtained using the DBSCAN clustering [50]. Finally, we were in position of using our GaussianFinder-specific parser to generate the CavBench’s XML file that describes all cavities of each protein.

Recall that all these geometric methods to detect cavities are agnostic to the type of cavities. In other words, they only deliver cavities, no matter if they are clefts, tunnels, or pores. According to the XML schema shown in Fig 2, agnostic cavities own the type NOTYPE. In fact, CavBench carries out the XML-based standardization of the ground-truth and method-specific cavities to make them comparable. That is, both ground-truth and method-specific cavities are expressed as clusters of spheres.

### Cavity benchmark

**Fig 5:**
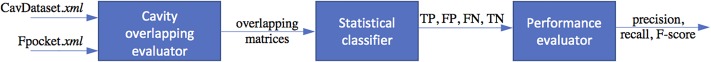
CavBench’s workflow to compare Fpocket-specific cavities with ground-truth cavities.

The cavity benchmark corresponds to the third workspace of CavBench, whose workflow is shown in Fig 5. Our cavity benchmark builds upon statistical classification (i.e., an example of supervised learning), as usual in machine learning and statistics. However, we are not using machine learning techniques as convolutional neural networks to determine cavities on protein surfaces. Nevertheless, we can see the ground-truth dataset as the training set, and the cavities determined by each method (e.g., Fpocket) as the testing set. Recall that our ground-truth consists of two subsidiary datasets. The first concerns the surface cavities (i.e., PDBsum clefts), while the second includes tubular cavities (i.e., PDBsum/Mole tunnels and pores). On the other hand, the cavities outputted by each geometric method are type-agnostic.

#### Cavity overlapping evaluator

To evaluate the prediction quality of putative binding-sites of each cavity detection method in the benchmark, we determine the Jaccard index (or Jaccard similarity coefficient) which measures similarity between method-specific cavities *c*_*i*_ (*i* = 1, …, *m*) and their ground-truth cavities *C*_*j*_ (*j* = 1, …, *n*). Given the pair (*c*_*i*_, *C*_*j*_), the Jaccard index *J*(*c*_*i*_, *C*_*j*_) is defined as the size of the intersection of such cavities divided by the size of their union, i.e.,

$$\begin{array}{c} J\left(c_{i},C_{j}\right)=\frac{c_{i}\cap C_{j}}{c_{i}\cup C_{j}}. \end{array}$$

Ideally, *J* = 1, i.e., *c*_*i*_ is congruent with *C*_*j*_. Thus, the Jaccard index works as a cavity similarity evaluator (or cavity overlapping evaluator) that computes the percentage of intersection relative to the union of *c*_*i*_ and *C*_*j*_. This amounts to determine the percentage of pseudo-atom centers of *c*_*i*_ ∩ *C*_*j*_ relative to pseudo-atom centers of *c*_*i*_ ∪ *C*_*j*_. Note that our overlapping condition given by the Jaccard index is stricter than the spanning condition used in other studies [53–55], which states that a prediction is a hit if the barycenter of the predicted pocket is within 4Å to any atom belonging to the ligand.

**Fig 6:**
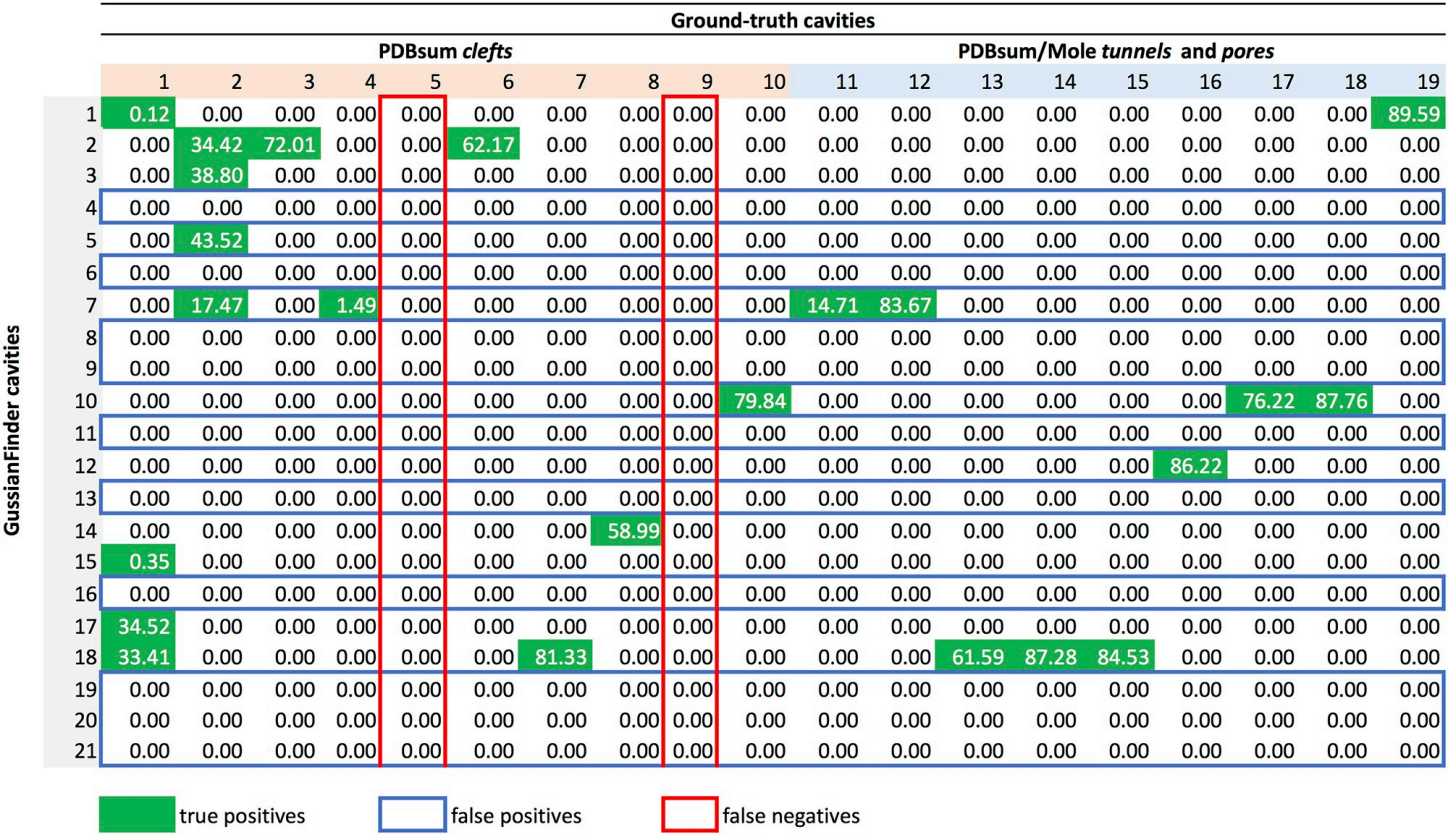
The overlapping matrix produced by the cavity overlapping evaluator for the protein 180L, as a result of benchmarking the cavities outputted by the GaussianFinder against the ground-truth dataset. Columns correspond to ground-truth cavities and rows to cavities detected by the GaussianFinder method. True positives (TP) correspond to rows (GaussianFinder cavities) containing at least a green cell; false positives (FP) are identified by rows (GaussianFinder cavities) without any green cell, i.e., they do not meet any ground-truth cavity; false negatives (FN) are identified by columns (i.e., ground-truth cavities) in red. This example contains 11 TP (rows), 10 FP (rows) and 2 FN (columns).

**Fig 7:**
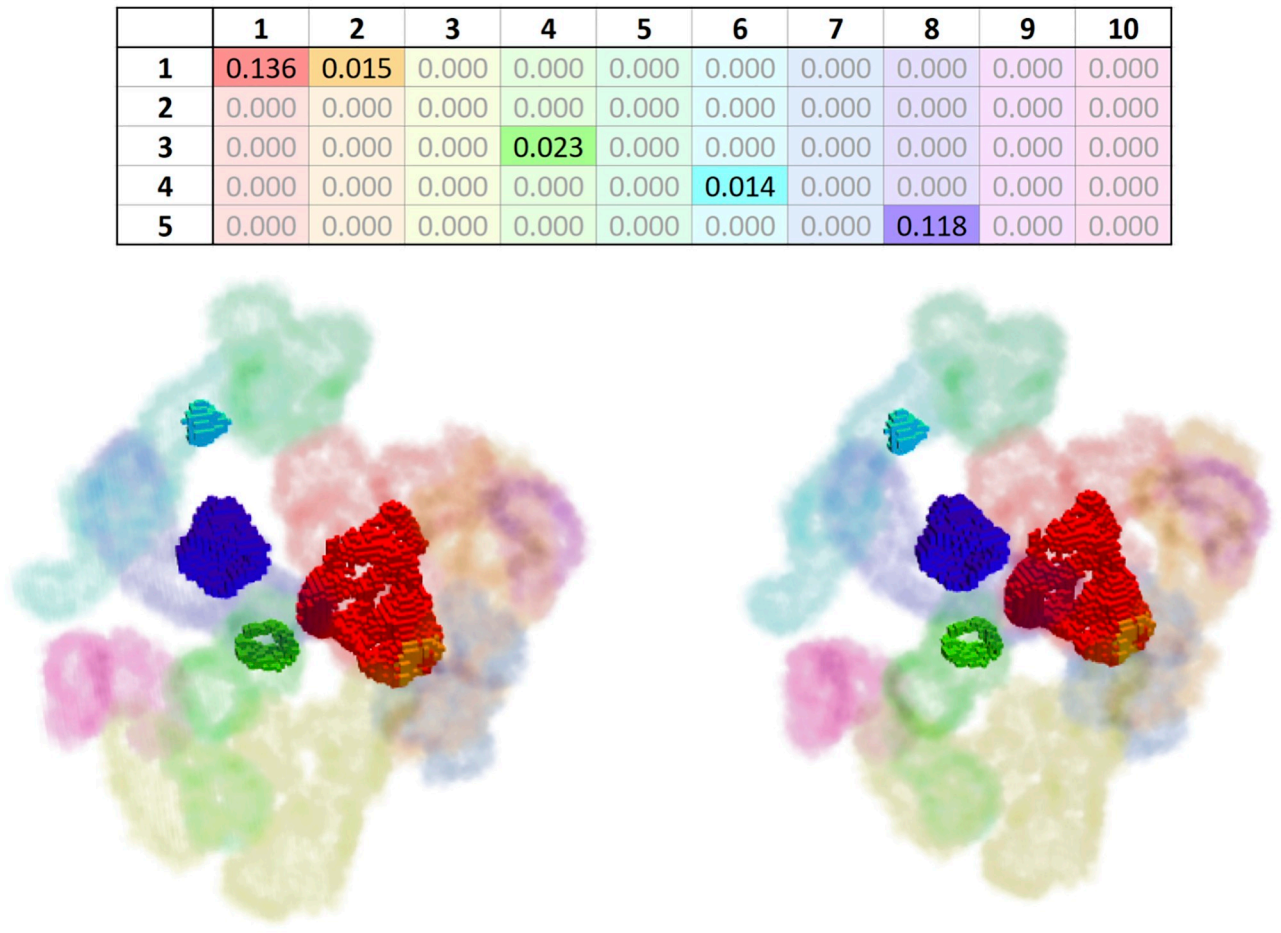
Overlapping matrix (top) and voxelized, *cross-eyed* stereoscopic 3D visualization of the protein 1A4U. The example represents the overlap between 5 method-detected cavities (rows) and 10 ground-truth cavities (columns). The overlapped portions of the ground-truth cavities are rendered with opaque colors (true positives), and the non-overlapped portions as semi-transparent (false negatives). This example shows that the method detected roughly 13.6% of the first cavity (red), 1.5% of the second (orange), 2.3% of the fourth (light green), 1.4% of the sixth (cyan), and 11.8% of the eigth (purple).

Thus, our cavity overlapping evaluator produces an *overlapping matrix* for each protein, as shown in Fig 6, where rows represent method-specific cavities and columns the ground-truth cavities; the first ten columns concern PDBsum clefts, and the remaining columns indicate PDBsum/Mole tunnels and pores. Taking into consideration that CavBench’s dataset includes 2293 proteins, we need to produce 2293 overlapping matrices to evaluate the performance of each cavity detection method. A 3D representation of part of an overlapping matrix depicting the overlapped volumes between cavities detected by the method and those from the ground-truth can be seen in Fig 7.

#### Statistical classifier

As illustrated in Fig 5, the statistical classifier computes the number of true positives (TP), false positives (FP), and false negatives (FN) from the overlapping matrix of each protein. A *true positive* is a hit, that is, a cavity *c*_*i*_ detected by a specific method that overlaps at least one ground-truth cavity *C*_*j*_ of a given protein, i.e., *c*_*i*_ ∩ *C*_j_ ≠ ⌀. For example, in Fig 6, we see that the cavity *c*_14_ detected by GaussianFinder for the protein 180L is a true positive because its Jaccard similarity index relative to the ground-truth cleft *C*_8_ is 58.99%. Furthermore, *c*_14_ does not overlap any other ground-truth cavity, so there is no repetition to consider. On the contrary, *c*_2_ overlaps three ground-truth cavities, namely the clefts *C*_2_, *C*_3_, and *C*_6_, in a scenario with repetitions. Obviously, discarding the repetitions, we would keep *C*_3_ because it is the most similar to *c*_2_, with a Jaccard index of 72.01%.

A *false positive* is a method-specific cavity that does not overlap any ground-truth cavity. For example, *c*_4_ in Fig 6 is a false positive because it does not intersect any ground-truth cavity. Therefore, we identify false positives by zeroed rows (only zeros) of the overlapping matrix or, equivalently, cavities detected by the method, but that do not exist in the ground-truth.

A *false negative* is a ground-truth cavity that is not detected by the method. For example, the cavity *C*_9_ is a false negative because it does not intersect any method-specific cavity for the given protein. False negatives can be recognized by zeroed columns, which denote cavities existing in the ground-truth, but not detected by a specific method.

The computation of *true negatives* (TN) is a bit more complex. A *true negative* is a protein cavity that is not part of the ground-truth nor is detected by a specific method for a given protein. This means that true negatives cannot be inferred from the overlapping matrix associated to a given protein. These true negative cavities are important because they can be seen as unknown binding sites, which eventually will be sites for binding new drugs or ligands. The procedure of computing true negatives for each method is as follows: (i) determine the convex hull of the protein; (ii) construct the union that comprises protein atoms, ground-truth cavity pseudo-atoms, and method-specific cavity pseudo-atoms; (iii) construct the difference between such convex hull (first step) and union (second step); (iv) apply DBSCAN to form undetected cavities from the empty difference space. Nevertheless, our statistical study focuses on positive (rather than negative) samples, so we end up not to compute TNs at all.

#### Performance evaluator

As illustrated in Fig 5, CavBench evaluates the performance of a given cavity detection method (e.g., Fpocket) through three metrics: precision (*p*), recall (*r*), and F-score (*F*).

The *precision* is a measure of the performance of a given cavity detection method. It is defined by the number of correctly identified cavities divided by the number of all identified cavities of a specific cavity detection method, that is:

$$\begin{array}{c} p=\frac{TP}{TP+FP} \end{array}$$ (1)

The *recall* is also known as sensitivity, true positive rate, or probability of detection, and is given by

$$\begin{array}{c} r=\frac{TP}{TP+FN} \end{array}$$ (2)

where *TP* + *FN* is the number of positive samples. In the context of protein cavity detection, the recall measures the rate (or percentage) of positives that are correctly identified by a specific method.

The previous two metrics (precision and recall) do not depend on the number of true negatives, that is, they both apply to the positive class. The *F-score* combines precision and recall metrics into a single performance metric for each cavity detection method. Specifically, the F-score is the harmonic mean of precision and recall [56], that is,

$$\begin{array}{c} F=\frac{2\;TP}{2\;TP+FP+FN} \end{array}$$ (3)

Thus, what determines whether a method performs better than another is its higher value of F-score. In fact, a brief glance at Tables 1 and 2 shows us that Fpocket and GaussianFinder perform better than the GHECOM and KVFinder because their F-score values are higher than those of the latter ones. Furthermore, Fpocket generally performs better than GaussianFinder, particularly when we do not consider repetitions.

**Table 1:** Classification (TP, FP, FN) and performance (*p*, *r*, *F*) results *with repetitions*. Four cavity detection methods are benchmarked against the ground-truth: Fpocket, GaussianFinder, GHECOM, and KVfinder.

Method	APOs						HOLOs						APOs + HOLOs		
	TP	FP	FN	*p*	*r*	*F*	TP	FP	FN	*p*	*r*	*F*	p	r	*F*
Fpocket	16722	1480	3644	0.92	0.82	0.86	50027	5722	8699	0.90	0.85	0.87	0.90	0.84	0.87
GaussianFinder	16441	2443	1251	0.87	0.93	0.90	45011	7939	2964	0.85	0.94	0.89	0.86	0.94	0.89
GHECOM	11875	5946	5307	0.67	0.69	0.68	33962	19566	13917	0.63	0.71	0.67	0.64	0.70	0.67
KVFinder	8074	1378	7590	0.85	0.52	0.64	23385	3643	19885	0.87	0.54	0.67	0.86	0.53	0.66

Abbreviations:

**APOs**: apo proteins; **HOLOs**: holo proteins.

**TP**: true positives; **FP**: false positives; **FN**: false negatives.

**p**: precision; **r**: recall; **F**: F-score.

**Table 2:** Classification (TP, FP, FN) and performance (*p*, *r*, *F*) results *without repetitions*. Four cavity detection methods are benchmarked against the ground-truth: Fpocket, GaussianFinder, GHECOM, and KVfinder.

Method	APOs						HOLOs						APOs + HOLOs		
	TP	FP	FN	*p*	*r*	*F*	TP	FP	FN	*p*	*r*	*F*	p	r	*F*
Fpocket	4697	4763	8291	0.50	0.36	0.42	13932	17522	20651	0.44	0.40	0.42	0.45	0.39	0.42
GaussianFinder	3774	4711	9214	0.44	0.29	0.35	9724	14793	24859	0.40	0.28	0.33	0.41	0.28	0.34
GHECOM	3629	8259	9359	0.31	0.28	0.29	10027	27233	24556	0.27	0.29	0.28	0.28	0.29	0.28
KVFinder	2608	2688	10380	0.49	0.20	0.29	7398	7985	27185	0.48	0.21	0.30	0.48	0.21	0.29

Abbreviations:

**APOs**: apo proteins; **HOLOs**: holo proteins.

**TP**: true positives; **FP**: false positives; **FN**: false negatives.

**p**: precision; **r**: recall; **F**: F-score.

## Results

As suggested by the CavBench workflow shown in Fig 5, we end up getting three types of results for each cavity detection method: overlapping matrices (one per protein), classification results, and performance results. These results can be all retrieved from CavBench since we provide the entire method-specific dataset (e.g., the set of all xxxx-GaussianFinder .xml files, one per protein, where xxxx denotes the alphanumeric identifier of each protein) beforehand. Optionally, results for a single protein or a small set of proteins can be also retrieved in the .csv file format.

### Overlapping results

By default, the overlapping matrices (one per protein) generated by CavBench for a cavity detection method provide raw results, that is, results with repetitions. In other words, not all method-specific cavities *c*_*i*_ establish a one-to-one relationship with ground-truth cavities *C*_*j*_.

A brief glance at Fig 6 shows us that the overlapping matrix concerning the protein 180L exhibits rows where a single method-detected cavity overlaps more than one ground-truth cavity. That is, overlapping results in Fig 6 include repetitions. For example, *c*_2_ intersects three ground-truth cavities, *C*_2_, *C*_3_, and *C*_6_; in this case, the repetitions (*c*_2_, *C*_2_) and (*c*_2_, *C*_6_) are thrown away (i.e., their overlapping percentages with *c*_2_ are set to zero) because *C*_3_ is the ground-truth cavity that most overlaps *c*_2_, with a percentage of 72.01%. Likewise, *C*_2_ is overlapped by four method-specific cavities, *c*_2_, *c*_3_, *c*_5_, and *c*_7_, but *c*_5_ is the one that most overlaps *C*_2_, with overlapping percentage of 43.52%; consequently, the overlapping percentages of (*c*_2_, *C*_2_), (*c*_3_, *C*_2_), and (*c*_7_, *C*_2_) are set to zero. Obviously, as explained below, removing repetitions alter the classification results in terms of TP, FP, and FN. Hence, we considered two scenarios for results, with and without repetitions (see Tables 1 and 2).

### Classification results

The classification results are expressed in terms of the number of TP, FP, and FN, and are obtained from the overlapping matrices associated with the proteins. Table 1 lists such results with repetitions for Fpocket, GaussianFinder, GHECOM, and KVFinder, while Table 2 presents the results without repetitions for the same four methods. By comparing the classification results of both Tables 1 and 2, we see that the removal of repetitions decreases the number of true positives, and increases the number of false positives and false negatives. For example, looking again at Fig 6, we see that *c*_5_ is a true positive because it is the the cavity that most overlaps or resembles *C*_2_; so, setting the overlapping percentage of (*c*_3_, *C*_2_) to zero originates a new false positive and, consequently, decrements the number of true positives. Furthermore, eliminating the repetitions may also lead to the increase of the number of false negatives and, at the same time, decreasing the number of true positives. For example, removing the repetition (*c*_7_, *C*_4_) creates a new false negative and decrements the number of true positives.

### Performance results

As usual, there are two methodologies to discuss the performance of a given method based on its results. The first methodology consists in finding *precision* values when the *recall* varies in the interval [0, 1], or vice-versa. The second methodology is more straightforward, and consists in combining both scores into a single measure. In either methodology, precision and recall scores are discussed jointly, not in isolation. Here, we have adopted the second methodology by computing the *F-score*, which, as mentioned above, is the harmonic mean of precision and recall.

The performance results are presented in Tables 1 and 2 considering both scenarios with and without repetitions, respectively. Note that performance results are better for the scenario with repetitions than without repetitions. For example, the F-score of GaussianFinder takes on the value of 0.89 considering repetitions, and 0.34 without repetitions. In fact, as seen above, discarding repetitions has the effect of reducing the number of true positives (TP) and increasing the number of false positives (FP) and false negatives (FN), while keeping the same total numbers of method-detected and ground-truth cavities, and thus changing the values of precision, recall, and F-score as given by Eqs (1)–(3).

#### Precision

In the scenario with repetitions (Table 1), Fpocket is more precise than any other method; that is, it is capable of correctly identifying more cavities (relative to all identified cavities) than the remaining methods. In fact, considering apo and holo proteins altogether, its precision is 0.90 (in the interval [0, 1]), while the precision values of GaussianFinder, GHECOM, and KVFinder are 0.86, 0.64, and 0.86, respectively. Note that Fpocket also performs more precisely than the other three methods in the scenario without repetitions (Table 2).

#### Recall

A brief glance at Tables 1 and 2 allows us to observe that the probability of a method to correctly detect positives is higher for the scenario with repetitions than without repetitions. In addition, such a probability (i.e., recall) is consistently higher for GaussianFinder than any other method. In fact, we see that GaussianFinder ranks first with a joint probability of detection (for apo and holo proteins) of 0.94, while Fpocket, GHECOM, and KVFinder come next with 0.84, 0.70, and 0.53, respectively. However, this ranking does not hold in the scenario without repetitions, as Fpocket performs better than any other method concerning recall, which takes on the value of 0.39.

#### F-score

By blending precision and recall into a single score, called F-score, we end up having only one metric to measure the performance of each cavity detection method. Based on the results shown in Tables 1 and 2 we see that GaussianFinder performs better than any other method in the scenario with repetitions (*F* = 0.89), while Fpocket ranks first in the scenario without repetitions (*F* = 0.42), considering apo and holo proteins as a whole.

### CavBench software

*CavBench* is available at https://github.com/mosqueteer/CavBench/. It was designed as a standalone software suite to run entirely on Linux, Mac OSX, and Windows (via Power Shell) using shell scripting. Its idea is to easily and quickly compare any new technique against state-of-the-art methods that detect cavities on protein surfaces. That is, CavBench is platform-independent.

Its platform-independence is reinforced by the fact that it does not run any protein cavity method. In fact, any new method should run outside the scope of CavBench, and produce its dataset of cavities concerning the 2293 proteins in the .xml or .csv formats, whose files are then processed by CavBench. Such an XML dataset describing cavities detected by a specific (eventually new) method allows us to compare it to other methods using information stored in CavBench. Essentially, the *CavBench* suite only runs the three programs in the workflow shown in Fig 5. Thus, the XML design of CavBench reinforces the interoperability between distinct operating systems and their versions. Besides, *CavBench* does not require installing or compiling programs in some remote execution environment. These features make the *CavBench* suite easy to run and its results more readily accessible to the research community.

## Conclusions

Identifying cavities on protein surfaces is an important step to both drug design and discovery, and protein docking. So far, researchers have used ad-hoc methods to compare each new cavity detection method against known binding sites. This has made it difficult to assess the relative strengths and limitations of new techniques, since there is no common ground against which to evaluate new research. To the best of our knowledge, no benchmark has been proposed in the literature to allow comparing cavity detection methods to one another or relative to a ground-truth dataset. We designed CavBench as such a benchmark to fill that gap.

As future work, we intend to also consider true negatives in our benchmark, as well as new metrics to account for the negative class. Furthermore, we plan to add more method-specific datasets (i.e., more methods) and ground-truth datasets to CavBench. We also plan to parallelize CavBench’s workflow using GPUs, as well as to create a graphical user interface and add more data visualization tools to provide a more friendly and interactive user experience while affording better insights into comparing method performances.
